# Supplementary figures and images for: White spot syndrome virus immediate-early protein (wsv100) antagonizes the NF-κB pathway to inhibit innate immune response in shrimp
Source: PLoS Pathog. 2025 Jun 12;21(6):e1012828. doi: 10.1371/journal.ppat.1012828 (PMC12187017; doi:10.1371/journal.ppat.1012828)

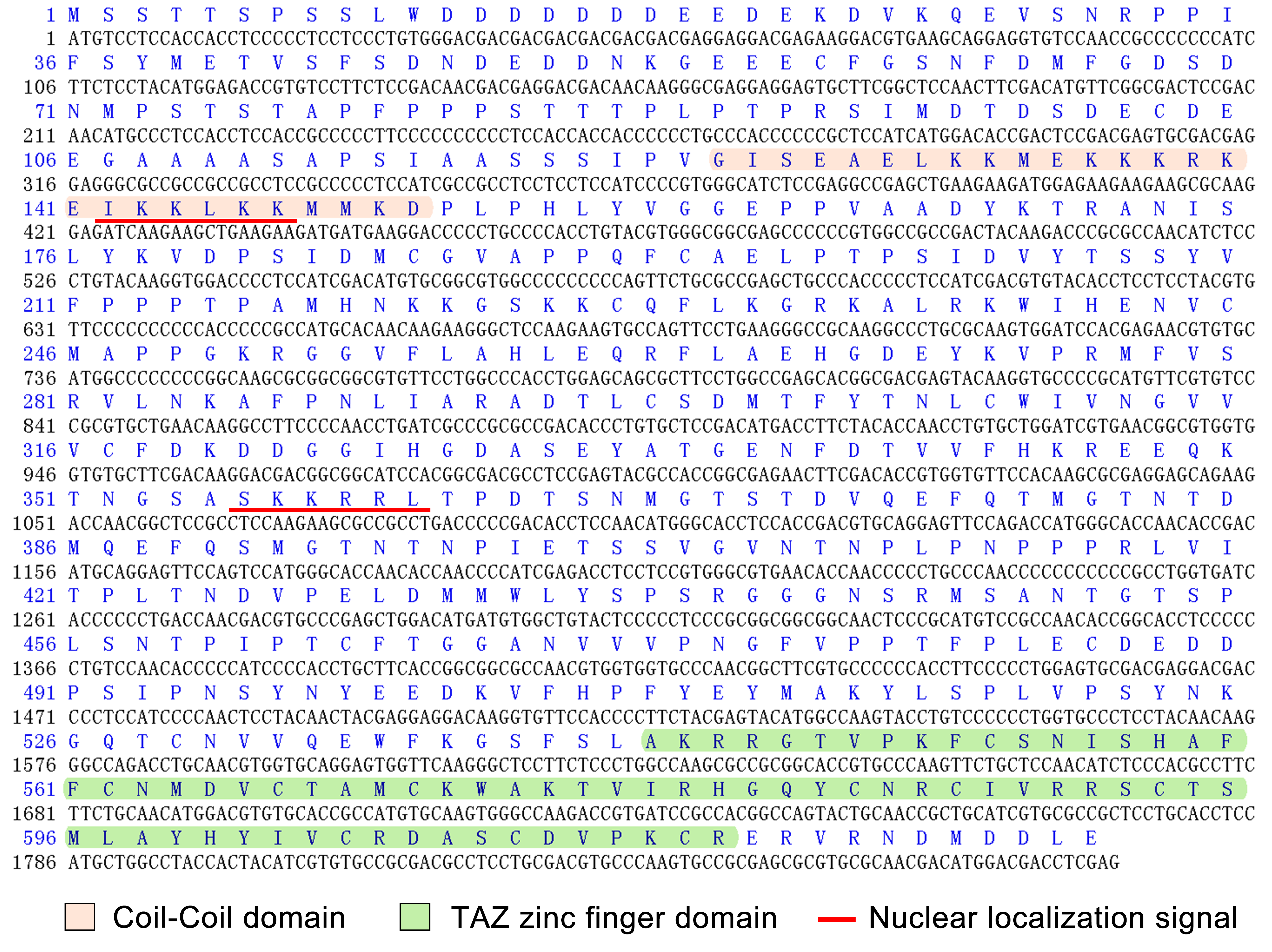

Supplement: S1 Fig — The orange represents coil-coil domain, the green represents TAZ zinc finger domain, and the red underline represents nuclear localization signal. (TIF) [file ppat.1012828.s001.tif]

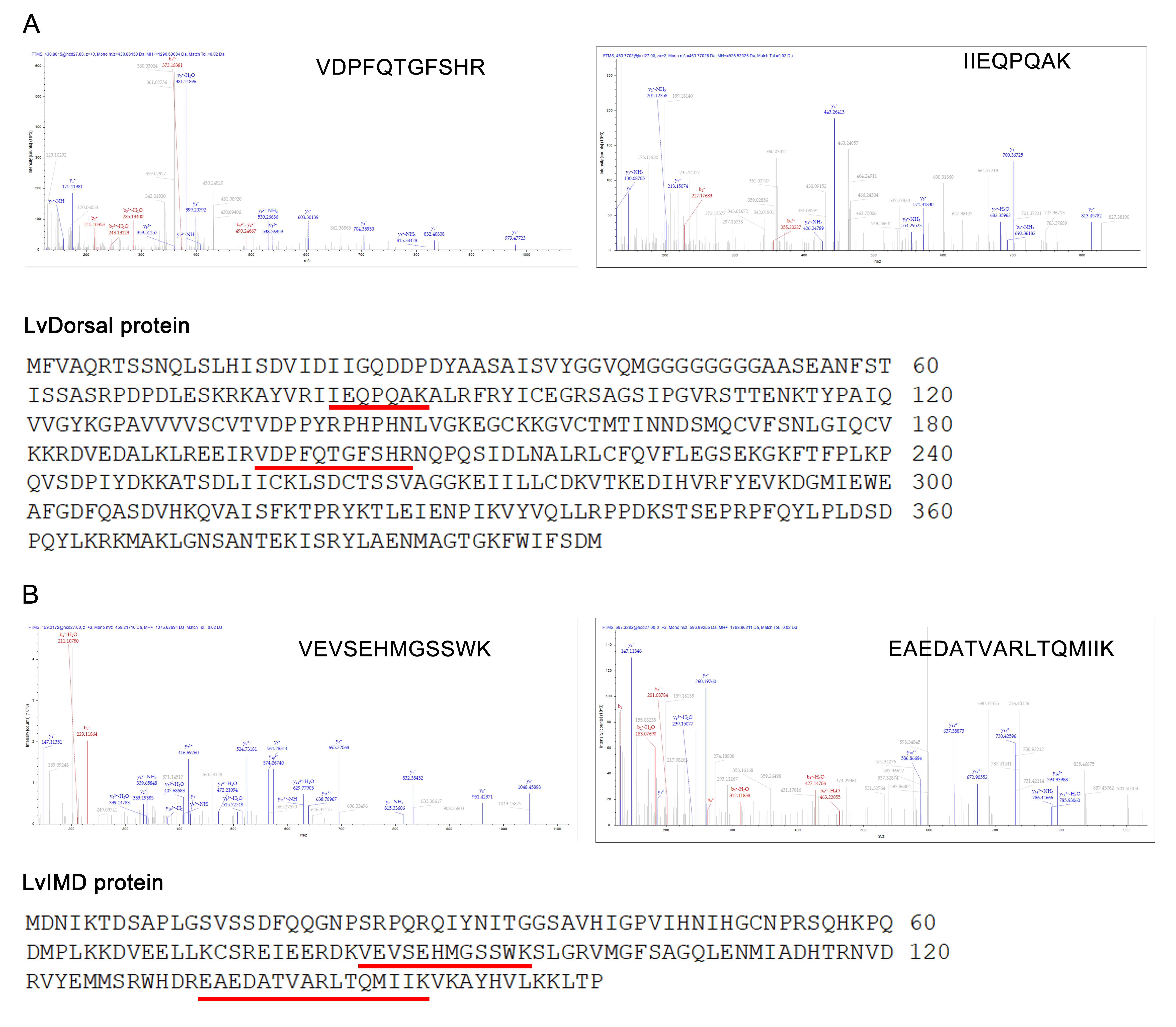

Supplement: S2 Fig — (A) MS/MS spectra were displayed for the identification of LvDorsal in the shrimp’s hemocyte lysates following His-tagged pulldown. Peptides identified through proteomic analysis are highlighted with underline, indicating their association with LvDorsal. (B) MS/MS spectra were displayed for the identification of LvIMD in the shrimp’s hemocyte lysates following His-tagged pulldown. Peptides identified through proteomic analysis are highlighted with underline, indicating their association with LvIMD. (TIF) [file ppat.1012828.s002.tif]

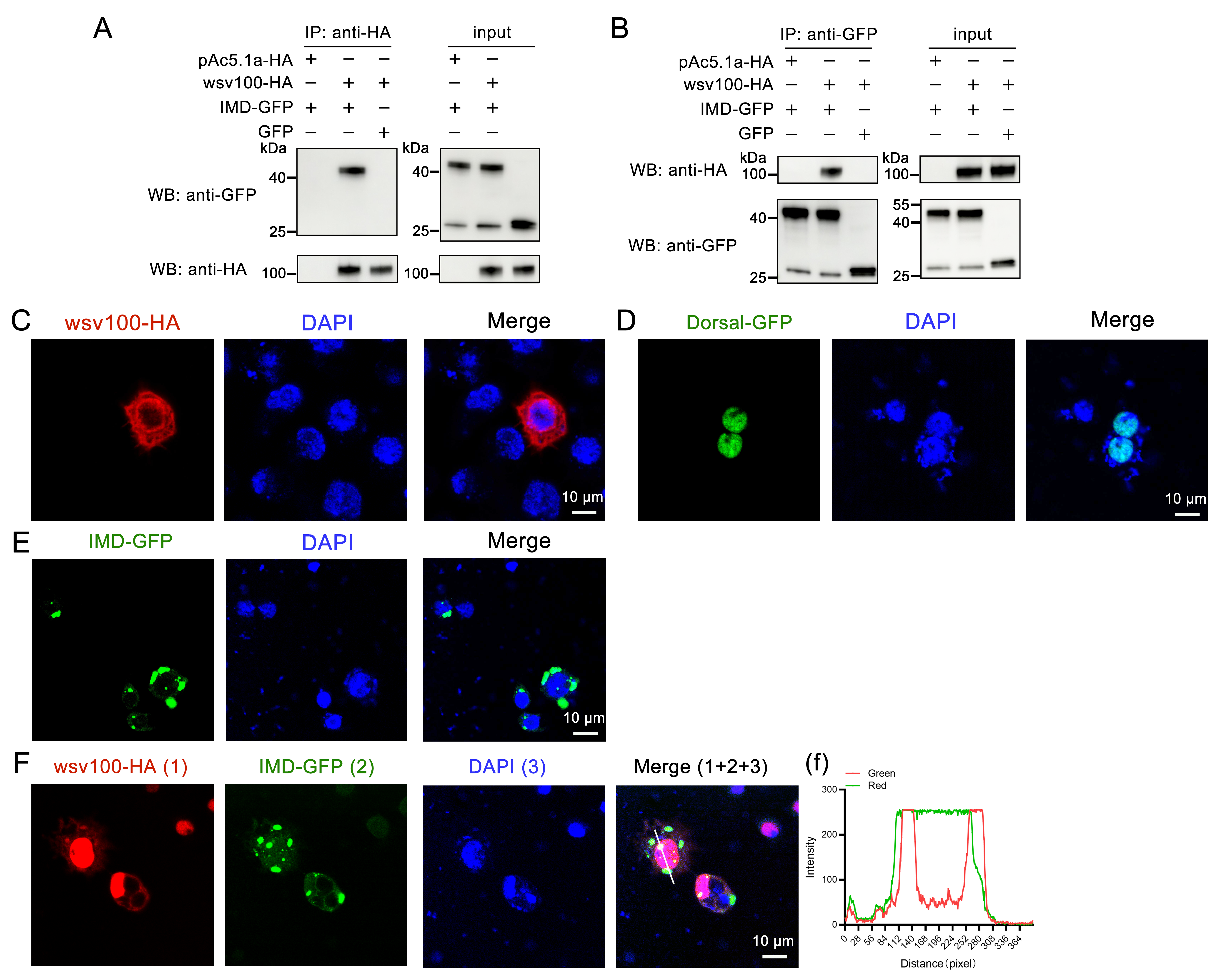

Supplement: S3 Fig — (A, B) The interaction between wsv100 and IMD were performed using CoIP. Proteins were expressed in S2 cells, and IP was conducted using anti-HA (A) or anti-GFP antibodies (B). Immunoprecipitates were detected with corresponding secondary antibodies. (C-E) The localization of wsv100 (C), Dorsal (D), and IMD (E) in S2 cells. (F) The colocalization of wsv100 with IMD in S2 cells. The wsv100 was detected with mouse anti-HA antibodies and anti-mouse Alexa Fluor 594. DAPI staining highlighted the nuclei. The scale bar represents 10 μm. (f) Quantitative analysis of fluorescence colocalization of wsv100 with IMD. Colocalization intensity was quantitatively analyzed, with complete colocalization indicated by overlapping peaks and maxima shifted by less than 20 nm. All experiments were representative of three biological replicates and yielded similar results. (TIF) [file ppat.1012828.s003.tif]

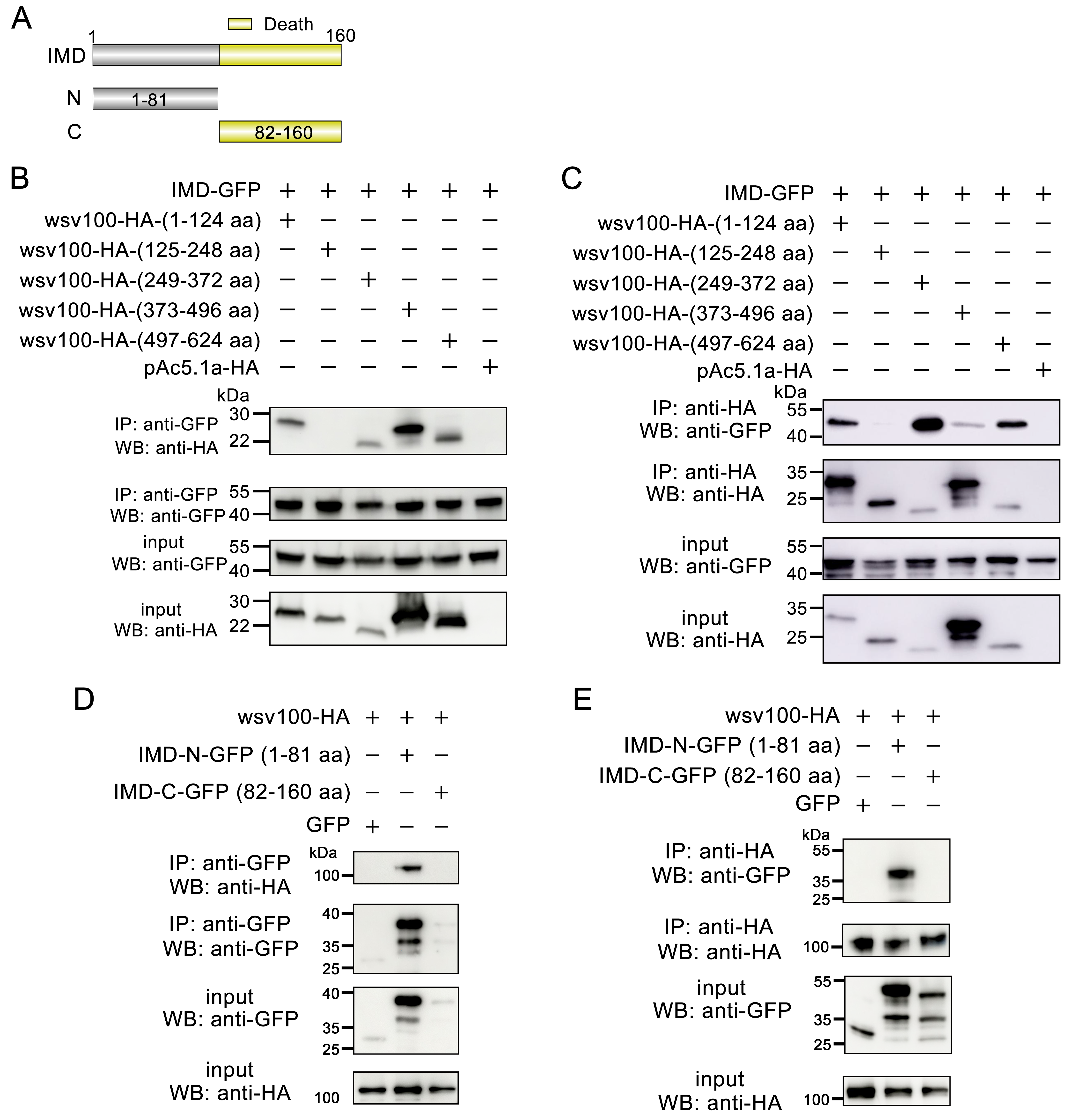

Supplement: S4 Fig — (A) Schematic diagram of IMD and its truncation mutants. (B-C) Identification of the wsv100 domain binding IMD. GFP-tagged IMD expressed in S2 cells were immunoprecipitated, and the presence of HA-tagged wsv100 truncation mutants were detected by GFP-immunoprecipitates (B) or HA-immunoprecipitates (C). Input samples were analyzed using anti-GFP and anti-HA antibodies. (D-E) Identification of the IMD domain binding to wsv100. HA-tagged wsv100 expressed in S2 cells was immunoprecipitated, and the presence of GFP-tagged IMD truncation mutants were detected by GFP-immunoprecipitates (D) or HA-immunoprecipitates (E). Input samples were analyzed using anti-GFP and anti-HA antibodies. Each experiment was conducted in triplicate, ensuring consistent and representative results. (TIF) [file ppat.1012828.s004.tif]

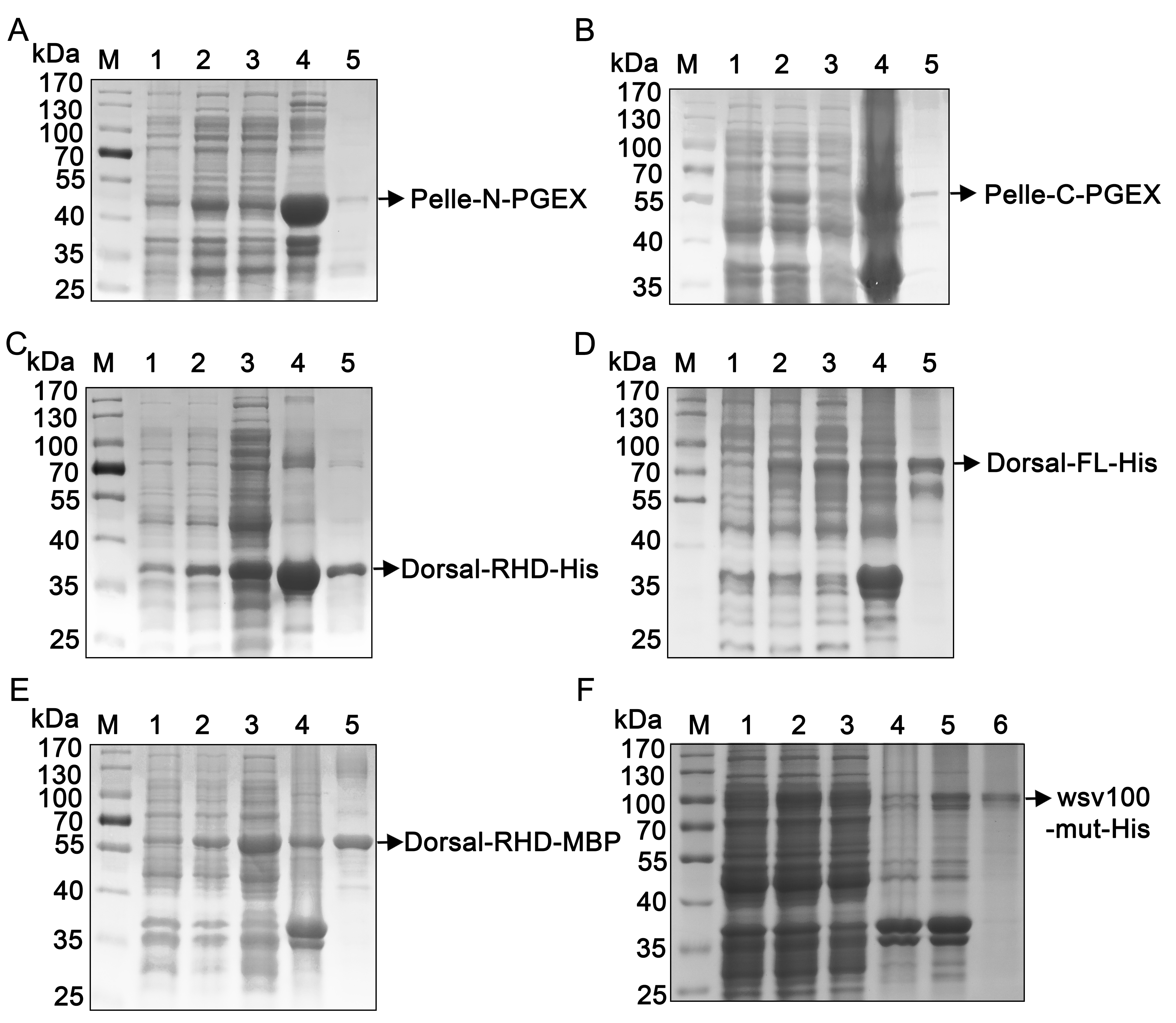

Supplement: S5 Fig — The recombinant protein expression and purification of Pelle-N-PGEX (A), Pelle-C-PGEX (B), Dorsal-RHD-His (C), Dorsal-FL-His (D), Dorsal-RHD-MBP (E), wsv100-mut-His (F). (TIF) [file ppat.1012828.s005.tif]
