# Supplementary material for: White spot syndrome virus immediate-early protein (wsv100) antagonizes the NF-κB pathway to inhibit innate immune response in shrimp
Source: PLoS Pathog. 2025 Jun 12;21(6):e1012828. doi: 10.1371/journal.ppat.1012828 (PMC12187017; doi:10.1371/journal.ppat.1012828)
Supplement: S1 Table — (DOCX) [file ppat.1012828.s006.docx]

**Supplementary table 1. Sequences of the primers used in this study.**

| **Primer** | **Sequence (5**'**-3**'**)** |
| --- | --- |

| **Protein expression** |
| --- |

| His-wsv100-F | ATGTCCTCGACGACCTCTCCAT |
| --- | --- |

| His-wsv100-R | TCAGTCATCCATGTCGTTGCGAA |
| --- | --- |
| His-Dorsal-RHD-F | ATGGTACGTATTATAGAACAGC |
| His-Dorsal-RHD-R | TTTGTCATAAATTGGATCTGAA |
| MBP-Dorsal-RHD-F | ATGGTACGTATTATAGAACAGC |
| MBP-Dorsal-RHD-R | TTTGTCATAAATTGGATCTGAA |
| His-Dorsal-FL-F | ATGGTACGTATTATAGAACAGC |
| His-Dorsal-FL-R | ATTATTAGCATTGCTTGGAGAC |
| GST-Pelle-N-F | ATGGCGCCTTGGCATAAGAAA |
| GST-Pelle-N-R | CAGAGCACAAGCACCAAGAGCA |
| GST-Pelle-C-F | ATGGCCACAGCTGTCTGGAACAC |
| GST-Pelle-C-R | TAAGGATATTTTTCTTGCTTGAGC |

| **Quantitative PCR** | |
| --- | --- |
| EF-1α-F | TATGCTCCTTTTGGACGTTTTGC |
| EF-1α-R | CCTTTTCTGCGGCCTTGGTAG |
| β-actin-F | CACGAGACCACCTACAACTCCATC |
| β-actin-R | TCCTGCTTGCTGATCCACATCTG |
| wsv100-F | ATTTTGCGCTGAATTGCCCA |
| wsv100-R | GGGCCATGCATACATTCTCG |
| VP28-F | AACACCTCCTCCTTCACCC |
| VP28-R | GGTCTCAGTGCCAGAGTAGGT |
| ALF1-F | GGATGTGGTGTCCTGGATGG |
| ALF1-R | GCGTCGTCCTCCGTGATG |
| ALF2-F | GCGAACAAACTCACTGGACTG |
| ALF2-R | ACATGCGACCCTGGAATACAG |
| ALF3-F | GACCTGTCCAACCCTGAGC |
| ALF3-R | TCGCCTCCTCCTCCGTTATC |
| ALF4-F | CCTGGTGGCACTCTTCGC |
| ALF4-R | ACGGTGAAGCGGCACTTATG |
| LYZ1-F | TACGCGACCGATTACTGGCTAC |
| LYZ1-R | AGTCTTTGCTGCGACCACATTC |
| LYZ2-F | CCCATGTTCCGATCTGATGTC |
| LYZ2-R | CACTTGCTGTTGTAAGCCACC |
| LYZ3-F | GTAGAAGATTGGAAATGTAACGAAGA |
| LYZ3-R | TGTGAATATGCCGTAGTCAAGG |
| LYZ4-F | ACGATGGAAGGGCAAAGGAG |
| LYZ4-R | AATAGGCAACACTTGATACTGAATGG |
| Pelle-F | CCACAAGTGCCACAGATGCA |
| Pelle-R | TGGCTGATCCGCGAATTCCCA |
| **Absolute quantitative PCR** | |
| WSSV32678-F | TGTTTTCTGTATGTAATGCGTGTAGGT |
| WSSV32678-R | CCCACTCCATGGCCTTCA |
| TaqMan probe-WSSV32706 | CAAGTACCCAGGCCCAGTGTCATACGTT |
| **RNAi** | |
| dsGFP-F | ATGGTGAGCAAGGGCGAGGAG |
| dsGFP-R | TTACTTGTACAGCTCGTCCATGCC |
| dsGFP-T7-F | GGATCCTAATACGACTCACTATAGGATGGTGAGCAAGGGCGAGGAG |
| dsGFP-T7-R | GGATCCTAATACGACTCACTATAGGTTACTTGTACAGCTCGTCCATGCC |
| dswsv100-F | CCCTCCTCAATTTTGCGCTG |
| dswsv100-R | ACGCCTCTTCTTACTGGCAC |
| dswsv100-T7-F | GGATCCTAATACGACTCACTATAGGCCCTCCTCAATTTTGCGCTG |
| dswsv100-T7-R | GGATCCTAATACGACTCACTATAGGACGCCTCTTCTTACTGGCAC |
| dsPelle-F | TCTCGTGGAGTGATTTGGATG |
| dsPelle-R | ATTTACCATTGCTTGCTTACGC |
| dsPelle-T7-F | GGATCCTAATACGACTCACTATAGTCTCGTGGAGTGATTTGGATG |
| dsPelle-T7-R | GGATCCTAATACGACTCACTATAGGATTTACCATTGCTTGCTTACGC |
| **Plasmid construction** | |
| wsv100-HA-F | ATGTCCTCCACCACCTCCCCCTC |
| wsv100-HA-R | GTCGTCCATGTCGTTGCGCACGC |
| wsv100-(1-124 aa)-HA -F | ATGTCCTCCACCACCTCCCC |
| wsv100-(1-124 aa)-HA -R | CACGGGGATGGAGGAGGAG |
| wsv100-(125-248 aa)-HA -F | GGCATCTCCGAGGCCGAGC |
| wsv100-(125-248 aa)-HA -R | GGGGGCCATGCACACGTTC |
| wsv100-(249-372 aa)-HA -F | CCCGGCAAGCGCGGC |
| wsv100-(249-372 aa)-HA -R | GGTGGAGGTGCCCATGTTGG |
| wsv100-(373-496 aa)-HA -F | GACGTGCAGGAGTTCCAGAC |
| wsv100-(373-496 aa)-HA -R | GGAGTTGGGGATGGAGGGG |
| wsv100-(497-624 aa)-HA -F | TACAACTACGAGGAGGACAAGGTG |
| wsv100-(497-624 aa)-HA -R | GTCGTCCATGTCGTTGCGC |
| Dorsal-GFP-F | ATGTTTGTTGCCCAGCGTACTTC |
| Dorsal-GFP-R | CATATCAGAAAATATCCAAAACTT |
| Dorsal-GFP-S1-F | ATGTTTGTTGCCCAGCGTACTTC |
| Dorsal-GFP-S1-R | ATAGGCTTTACGTTTGCTCTCCA |
| Dorsal-GFP-S2-F | ATGGTACGTATTATAGAACAGC |
| Dorsal-GFP-S2-R | TTTGTCATAAATTGGATCTGAA |
| Dorsal-GFP-S3-F | ATGAAGGCCACATCAGATCTGA |
| Dorsal-GFP-S3-R | TCACATATCAGAAAATATCCAAA |
| IMD-GFP-F | ATGGATAATATTAAGACAGATTC |
| IMD-GFP-R | TCAAGGTGTAAGCTTCTTCAGCA |
| IMD-GFP-N-F | ATGGATAATATTAAGACAGATTC |
| IMD-GFP-N-R | TCGTTCCTCGATCTCGCGACTG |
| IMD-GFP-C-F | ATGGACAAGGTCGAGGTCAGC |
| IMD-GFP-C-R | TCAAGGTGTAAGCTTCTTCAGCA |
